# Blood and serum samples collection and storage for further selenium measurements

**DOI:** 10.1186/1897-4287-10-S3-A16

**Published:** 2012-04-20

**Authors:** Magdalena Muszyńska, Grzegorz Sukiennicki, Anna Jakubowska, Jan Lubiński

**Affiliations:** 1 Pomeranian Medical University, Szczecin, Poland and Read-Gene SA, Szczecin, Poland

## 

The aim of the study was to determine the right storage and transport conditions for blood and serum samples intended for later selenium measurements. Blood and serum samples were stored in different temperature conditions (+4°C and -20°C). After 3, 7 and 14 days the selenium level was measured in each sample and the results were compared with the initial selenium level. Selenium was quantitatively measured using graphite furnance atomic absorption spectrometry (GF-AAS) with Zeeman correction (AAnalyst 600, PerkinElmer).

Storage of whole blood samples in -20°C occurred to be inappropriate. Centrifugation of serum for the measurement was not possible even after the shortest time of incubation. However, samples stored at +4°C were measured and analyzed. After the period of 3, 7 and 14 days the selenium level in the sample was higher than initially (7%, 11% and 24% respectively). On the other hand, neither the temperature nor the time of storage of centrifuged serum samples had any influence on selenium level. In all analyzed cases the selenium level did not differ more than 10% of initial level.

The experiment clearly showed how blood or serum samples should be stored before the measurement to obtain correct and repeatable results of selenium level. The experiment will be also very helpful in creation of a set of rules for laboratories in different places in Poland and Europe for proceeding with samples after collection to assure the highest quality of results.

